# Translocator Protein (TSPO) as a Potential Biomarker in Human Cancers

**DOI:** 10.3390/ijms19082176

**Published:** 2018-07-25

**Authors:** Nimisha H. Bhoola, Zukile Mbita, Rodney Hull, Zodwa Dlamini

**Affiliations:** 1 Faculty of Health Sciences, University of the Witwatersrand, Johannesburg 2193, South Africa; nimisha.bhoola@wits.ac.za; 2 Department of Biochemistry, Microbiology and Biotechnology, University of Limpopo, Private Bag X1106, Sovenga 0727, South Africa; Zukile.Mbita@ul.ac.za; 3 Research, Innovation & Engagements Portfolio, Mangosuthu University of Technology, Durban 4031, South Africa, rodneyhull@gmail.com

**Keywords:** *Tspo*, prostate cancer, stomach cancer, colon cancer, liver cancer, lung cancer, kidney cancer, breast cancer, brain cancer

## Abstract

TSPO is a receptor involved in the regulation of cellular proliferation, apoptosis and mitochondrial functions. Previous studies showed that the expression of TSPO protein correlated positively with tumour malignancy and negatively with patient survival. The aim of this study was to determine the transcription of *Tspo* mRNA in various types of normal and cancer tissues. In situ hybridization was performed to localise the *Tspo* mRNA in various human normal and cancer tissues. The relative level of *Tspo* mRNA was quantified using fluorescent intensity and visual estimation of colorimetric staining. RT-PCR was used to confirm these mRNA levels in normal lung, lung cancer, liver cancer, and cervical cancer cell lines. There was a significant increase in the level of transcription in liver, prostate, kidney, and brain cancers while a significant decrease was observed in cancers of the colon and lung. Quantitative RT-PCR confirmed that the mRNA levels of *Tspo* are higher in a normal lung cell line than in a lung cancer cell line. An increase in the expression levels of *Tspo* mRNA is not necessarily a good diagnostic biomarker in most cancers with changes not being large enough to be significantly different when detected by in situ hybridisation.

## 1. Introduction

The translocator protein (TSPO), previously known as the peripheral benzodiazepine receptor [1], is an 18 kDa evolutionary conserved protein that was initially described as a binding site for benzodiazepine drugs outside of the central nervous system (CNS), but was later found to be expressed in every mammalian organ [2,3,4]. The name of the protein was changed to reflect the role of this receptor in the transport of cholesterol across the mitochondrial membrane, the first and rate-limiting step of steroidogenesis, as well as to reflect the putative roles in protein import, and in porphyrin binding and transport [5,6,7,8,9,10,11,12]. Despite its embryonic development and cholesterol transporter functions being central to our understanding of the physiological role of TSPO, these roles have recently been challenged [13,14,15]. Regardless of this a cholesterol recognition/interaction amino acid consensus (CRAC) sequence was identified as the cholesterol binding site based on an NMR structure of mouse TSPO [13]. *Tspo* knockout studies have consistently revealed that the absence of TSPO results in altered mitochondrial energy metabolism, together with lower oxygen consumption, membrane potential, and ATP levels. TSPO is also thought to be involved in Parkinson’s and Alzheimer’s diseases, inflammation, and tumour progression [13].

Sub-cellularly, the TSPO protein is localized to the outer mitochondrial membrane and is associated with the 32 kDa voltage-dependent anion channel (VDAC) and 30 kDa adenine nucleotide carrier (ANC) to form a trimeric complex [3,16]. However, it may also be found on the plasma membrane of some cells such as erythrocytes, which are devoid of mitochondria [17], in the non-mitochondrial fractions of hepatocytes [18] and in and around the nucleus of cells of human breast tumour biopsies and breast tumour cell lines [19].

TSPO protein expression varies throughout the body, ranging from high in steroidogenic tissues [3,4,20], to moderate in renal tissues, the reproductive organs, and lungs [20,21,22], to low in the liver and specific areas of the brain [5,21,23]. An increase in the expression of the TSPO protein has been seen in a wide variety of malignant human cells and tissues including brain cancers [24,25], prostate cancers [26], colon cancers [27,28,29], breast cancers [30,31], oesophageal cancers [32], endometrial carcinomas [33], ovarian cancers, and hepatic carcinomas [34]. Furthermore, TSPO protein expression correlated positively with disease progression of some cancers, including oral cancers [35], brain cancers [24], colon cancers [29] and breast cancers [19,30]. The prognosis of some cancers also correlates negatively with the expression of TSPO [29,36,37].

The function, pharmacology and expression of the TSPO protein is well established and has been especially well studied with regard to drug binding interactions. Comprehensive reviews regarding this subject are available [38]. Despite this, the genetic regulation and mRNA expression patterns of *Tspo* are not well established and the complex mechanism where TSPO expression is regulated is not completely understood. Therefore, the aim of this study was to determine the expression of *Tspo* mRNA in various types of human normal and cancer tissues. This study uniquely examined the novel use of mRNA to examine the transcript levels of *Tspo* to diagnose cancer and compare *Tspo* across cancer in multiple tissues.

## 2. Results

### 2.1. Tspo mRNA Transcription Is Observed within Specific Cell Types in Normal and Cancer Tissues of Different Organs

*Tspo* mRNA transcription was observed in all human normal and cancer tissues examined. As expected, *Tspo* mRNA localization was observed to mainly occur in the cytoplasm. However, nuclear localization of the *Tspo* mRNA occurred in some cases. A summary of the changes in cellular localization that occur in various cancers is detailed in Table 1.

#### 2.1.1. Prostate

**Figure 1:**
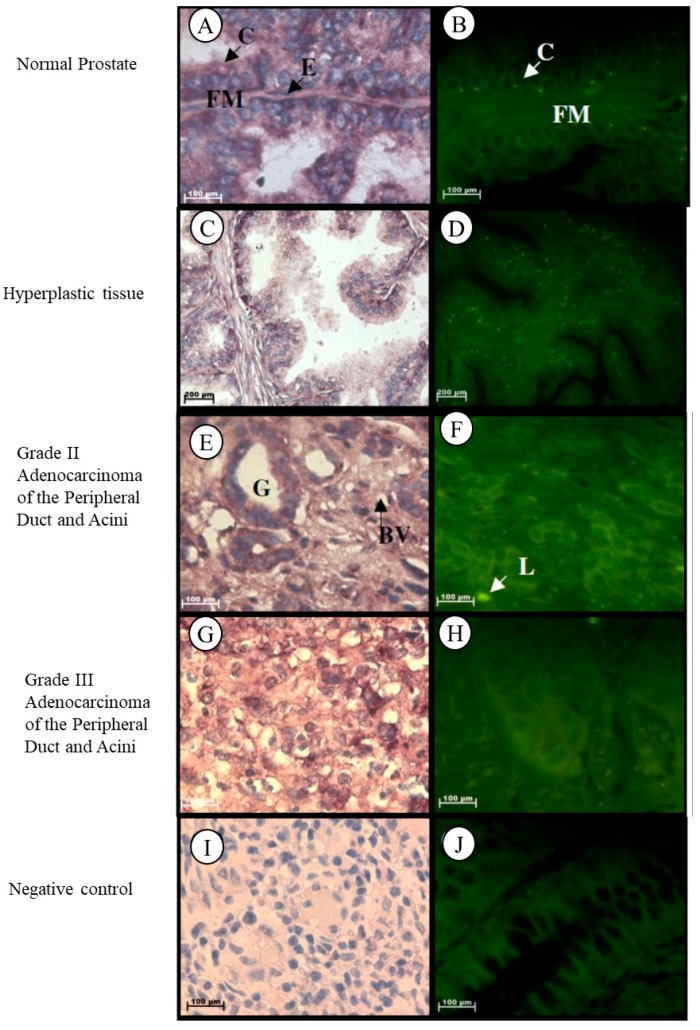
Localization of *Tspo* mRNA in healthy and diseased prostate tissues: In normal tissue (**A**,**B**) *Tspo* mRNA is localized to the cytoplasm of the inner columnar epithelium (**C**) and in the cytoplasm and elastic fibres (FM) and in the nuclei of fibroblasts of the fibromuscular stroma. Transcription of *Tspo* increases in hyperplastic tissue, (**C**,**D**) as shown by the increase in fluorescence intensity (**D**). In Grade II adenocarcinoma (**E**,**F**) *Tspo* mRNA is localized within the cytoplasm of cuboidal to low columnar epithelial cells lining glands (G), in the collagen fibres located in the stroma and in the nuclei and cytoplasm of lymphocytes (L) and endothelial cells lining blood vessels located in the surrounding stroma. In Grade III adenocarcinoma (**G**,**H**) Tspo mRNA is found in the cytoplasm of tumor cells that grow in sheets. Negative control for the subcellular localization of *Tspo* mRNA (**I**,**J**). Sense probes were used to probe tissue preparations. Tissues are magnified at 1000×.

In normal prostate tissue, *Tspo* mRNA is observed in the cytoplasm of the inner columnar epithelium that lines the glands and in the collagen and elastic fibres of the fibromuscular stroma, in the cytoplasm and nuclei of the outer cuboidal to flattened endothelial cells that line the glands and in the nuclei of fibroblasts of the fibromuscular stroma (Figure 1A,B). In hyperplastic prostate tissue, *Tspo* mRNA is found in the cytoplasm of the inner columnar epithelium and in the cytoplasm and nuclei of the outer cuboidal to flattened endothelial cells that line the glands, in the cytoplasm of the collagen and elastic fibres and in the nuclei of fibroblasts of the fibromuscular stroma (Figure 1C,D). *Tspo* mRNA was localized in the cytoplasm of cuboidal to low columnar epithelial cells lining glands and in the collagen fibres of the fibromuscular stroma and in the nuclei and cytoplasm of lymphocytes and endothelial cells lining blood vessels of the fibromuscular stroma of Grade II adenocarcinoma of the peripheral duct and acini (Figure 1E,F). Localization of the *Tspo* mRNA was found in the cytoplasm of tumour cells that grow in nests or sheets of Grade III adenocarcinoma of the peripheral duct and acini (Figure 1G,H).

#### 2.1.2. Stomach

**Figure 2:**
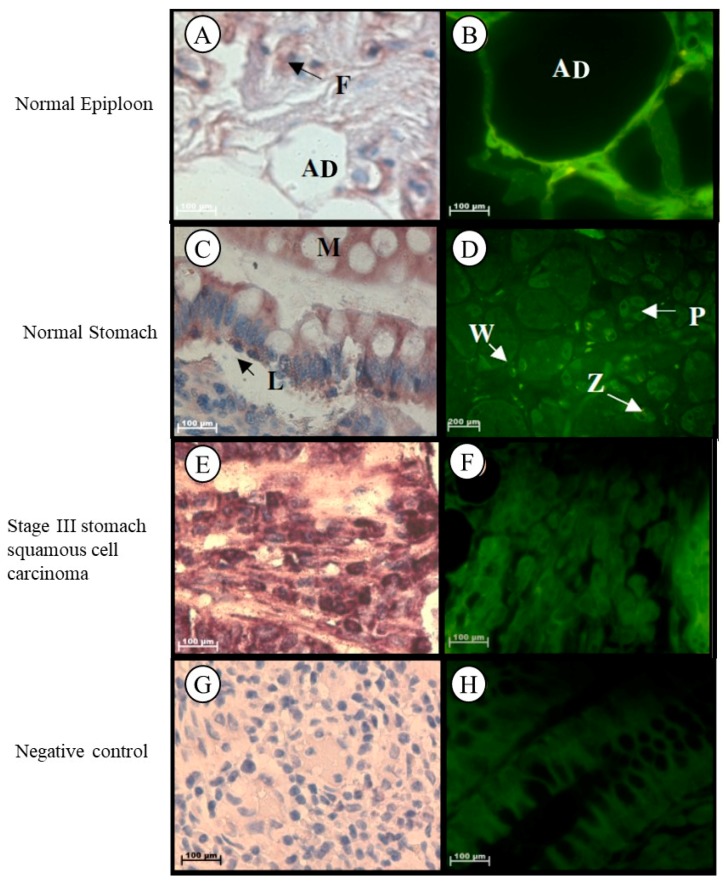
Subcellular Localization of *Tspo* mRNA in healthy and diseased stomach tissue: In normal epiploon tissue (**A**,**B**) *Tspo* mRNA is found in adipose cells (AD) as well as in fibroblasts (F). In healthy stomach Tissue (**C**,**D**) *Tspo* mRNA is localized in the cytoplasm of parietal cells (P), chief cells (Z), surface mucous cells (M) of the gastric glands and in the cytoplasm of plasma cells (P) and macrophages (W) and nuclei and cytoplasm of lymphocytes (L) located in the lamina propria. In stomach squamous cell. carcinoma (**E**,**F**) *Tspo* mRNA is localized in the cytoplasm of tumour cells and collagen fibres in the surrounding stroma. (**G**,**H**) Negative control for the subcellular localization of *Tspo* mRNA. Sense probes were used to probe tissue preparations and this was analysed colorimetrically and fluorescently. Tissues are magnified at 1000×.

Localization of *Tspo* mRNA was found in the cytoplasm of adipose cells, collagen fibres, and in fibroblasts located between the collagen fibres in the stroma of epiploon (Figure 2A,B). In normal stomach tissue, *Tspo* mRNA was observed in the cytoplasm of parietal cells, chief cells, in the surface and neck mucous cells of the gastric glands, in collagen fibres, plasma cells, and macrophages located in the lamina propria, as well as in the nuclei and cytoplasm of lymphocytes located in the lamina propria (Figure 2C,D). *Tspo* mRNA was localized in the tumour cells and collagen fibres in the surrounding stroma of Grade III stomach squamous cell carcinoma (Figure 2E,F).

#### 2.1.3. Colon

**Figure 3:**
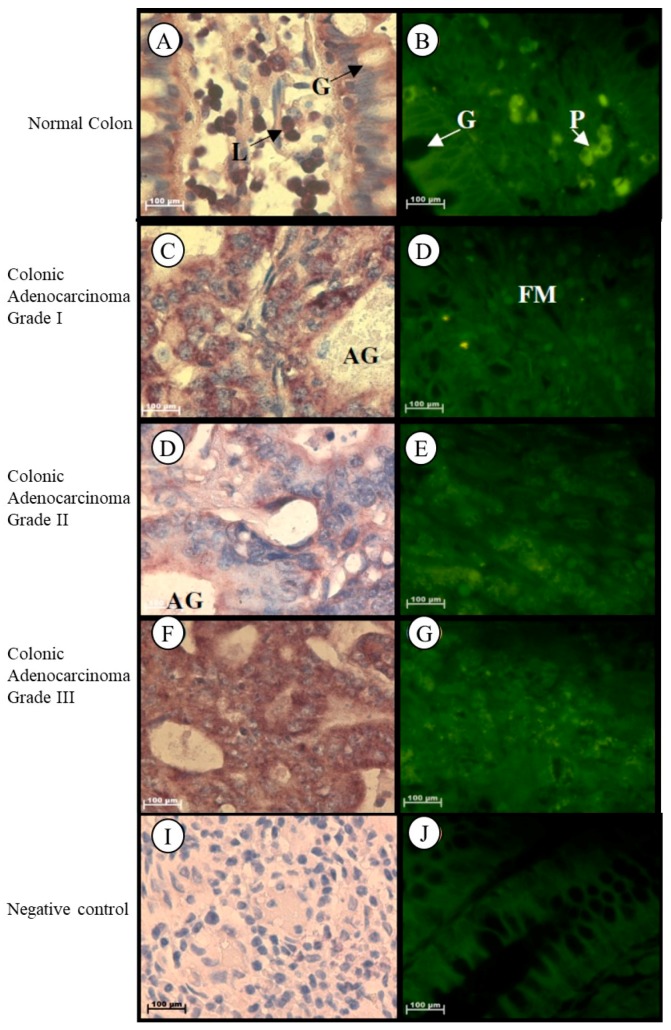
Localization of *Tspo* mRNA in healthy and diseased colon tissue. *Tspo* mRNA was localized in the cytoplasm of goblet (G) and absorptive cells of the Crypts of Lieberkhün and plasma cells (P) located in the lamina propria, and in the nuclei and cytoplasm of lymphocytes (L) located in the lamina propria of normal colon tissue (**A**,**B**). Localization of the *Tspo* mRNA was found in the cytoplasm of tumour cells that are mucin-secreting and arranged in adenomatous tubular glands (AG) and fibromuscular stroma (FM) of Grade I colonic adenocarcinoma (**C**,**D**). In Grade II colonic adenocarcinoma, *Tspo* mRNA was localized in the cytoplasm of tumour cells arranged as adenomatous glands (AG) or solid sheets (**E**,**F**). *Tspo* mRNA was localized in the cytoplasm of tumour cells that are predominantly arranged as a solid pattern of Grade III colonic adenocarcinoma (**G**,**H**). (**I**,**J**) Negative control for the subcellular localization of *Tspo* mRNA. Sense probes were used to probe tissue preparations and this was analysed colorimetrically and fluorescently. Tissues were magnified at 1000×.

In normal colon tissue *Tspo* mRNA was found in the cytoplasm of the goblet and absorptive cells of the Crypts of Lieberkhün, in the plasma cells located in the lamina propria, and in the nuclei and cytoplasm of lymphocytes located in the lamina propria of normal colon tissue (Figure 3A,B). Localization of the *Tspo* mRNA was found in the cytoplasm of tumour cells that are mucin-secreting and arranged in adenomatous tubular glands and fibromuscular stroma of Grade I colonic adenocarcinoma (Figure 3C,D). In Grade II colonic adenocarcinoma, *Tspo* mRNA was localized in the cytoplasm of tumour cells arranged as adenomatous glands or solid sheets (Figure 3E,F). *Tspo* mRNA was localized in the cytoplasm of tumour cells that are predominantly arranged as a solid pattern of Grade III colonic adenocarcinoma (Figure 3G,H).

#### 2.1.4. Liver

**Figure 4:**
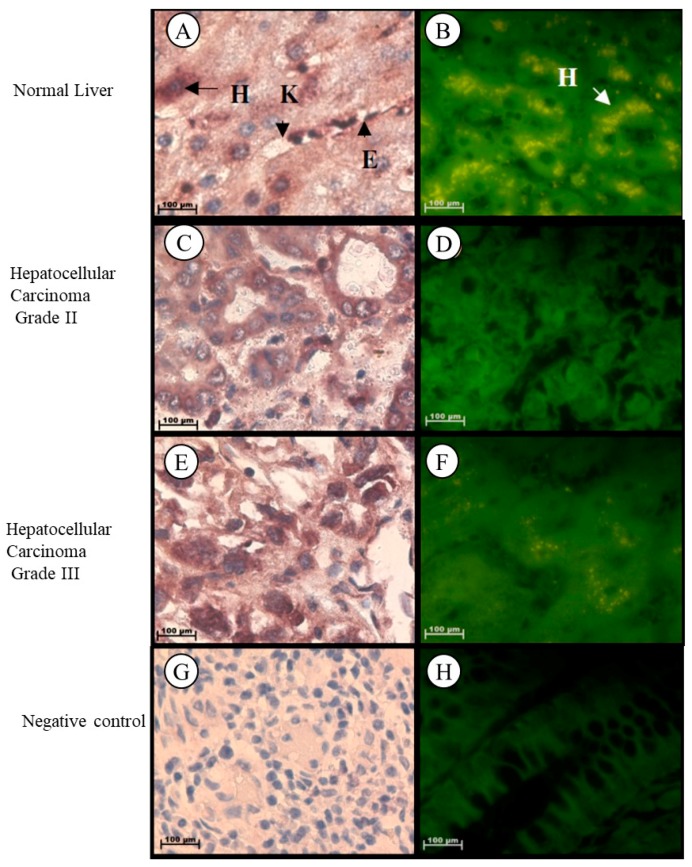
Subcellular Localization of *Tspo* mRNA in healthy and diseased Liver Tissues: Localization in Normal Liver Tissue (**A**,**B**) where *Tspo* mRNA is expressed in the cytoplasm of hepatocytes (H) and in the nuclei and cytoplasm of Kupffer cells (K) and endothelial cells (E) of the sinusoids. A comparison of the fluorescent intensity between cancer (**D**,**F**) and healthy tissue (**B**) shows a decrease in the expression level of *Tspo* in cancer. Grade II hepatocellular carcinoma (**C**,**D**) express PBR mRNA in the cytoplasm of tumour cells having either a trabecular or pseudoglandular pattern while Grade III hepatocellular carcinoma (**E**,**F**) express PBR mRNA in the nuclei and cytoplasm of tumour cells that resemble anaplastic giant cells to spindle-shaped cells. (**G**,**H**) Negative control for the subcellular localization of *Tspo* mRNA. Sense probes were used to probe tissue preparations and this was analysed colorimetrically and fluorescently. Tissues were magnified at 1000×.

In normal liver tissue, *Tspo* mRNA was observed in the cytoplasm of hepatocytes and in the nuclei and cytoplasm of endothelial cells of the sinusoids and Kupffer cells (Figure 4A,B). *Tspo* mRNA was localized in the cytoplasm of tumour cells having either a trabecular or pseudoglandular pattern of Grade II hepatocellular carcinoma (Figure 4C,D). Localization of the *Tspo* mRNA was found in the nuclei and cytoplasm of tumour cells that resemble anaplastic giant to spindle-shaped cells of Grade III hepatocellular carcinoma (Figure 4E,F).

#### 2.1.5. Lung

**Figure 5:**
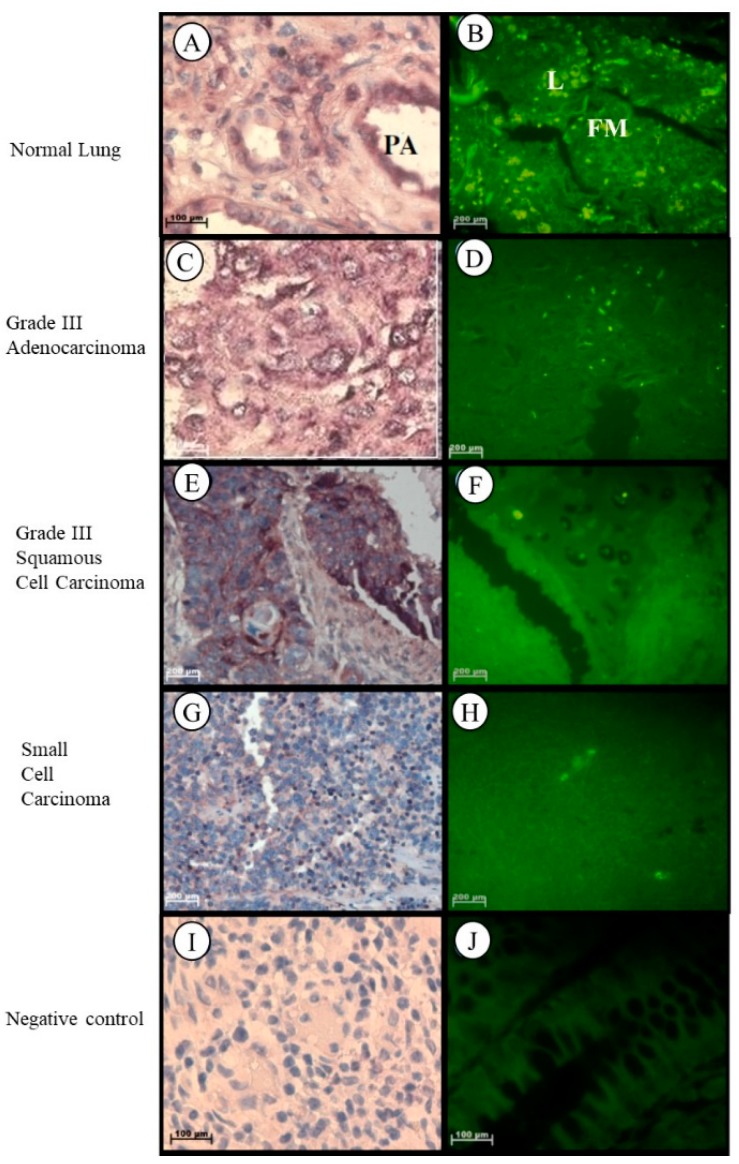
Subcellular Localizations of *Tspo* mRNA in healthy and diseased lung tissue: In healthy lung tissue (**A**,**B**) *Tspo* mRNA is expressed in the cuboidal cells that line the respiratory bronchiole in the smooth muscle fibres surrounding the pulmonary artery (PA), and in the nuclei and cytoplasm of the endothelial cells of the pulmonary artery and lymphocytes (L) in the surrounding stroma (FM). A comparison of the fluorescent intensity shows that there is no significant difference between *Tspo* expression in cancer (**D**,**F**,**H**) and normal lung (**B**) tissue. Grade III lung adenocarcinoma (**C**,**D**) expresses *Tspo* mRNA in the cytoplasm and nuclei of tumour cells and in the fibres located in the surrounding solid like stroma. In Grade III squamous cell carcinoma (**E**,**F**) *Tspo* mRNA is localized in the cytoplasm and nuclei of tumour cells. In small cell carcinoma (**G**,**H**) localization occurs in the cytoplasm of tumour cells that resemble lymphocytes and in the nuclei of lymphocytes and endothelial cells lining capillaries in the stroma. (**I**,**J**) Negative control for the subcellular localization of *Tspo* mRNA. Sense probes were used to probe tissue preparations and this was analysed colorimetrically and fluorescently. Tissues are magnified at 1000×.

In healthy lung tissue, *Tspo* mRNA was found in the cytoplasm of macrophages, plasma cells and fibroblasts in the surrounding stroma, in the cuboidal cells that line the respiratory bronchiole, in the smooth muscle fibres surrounding the pulmonary artery, and in the nuclei and cytoplasm of the endothelial cells of the pulmonary artery and lymphocytes in the surrounding stroma of healthy lung tissue (Figure 5A,B). In Grade III lung adenocarcinoma, *Tspo* mRNA was observed in the cytoplasm and nuclei of tumour cells and in the fibres located in the surrounding solid-like stroma (Figure 5C,D). *Tspo* mRNA was localized in the cytoplasm and nuclei of tumour cells of Grade III lung squamous cell carcinoma that is characterized by the merging of tumour cells to form a large cell pattern (Figure 5E,F). Localization of the *Tspo* mRNA in lung small cell carcinoma was found in the cytoplasm of tumour cells that resemble lymphocytes and in the nuclei of lymphocytes and endothelial cells lining capillaries in the stroma (Figure 5G,H).

#### 2.1.6. Kidney

**Figure 6:**
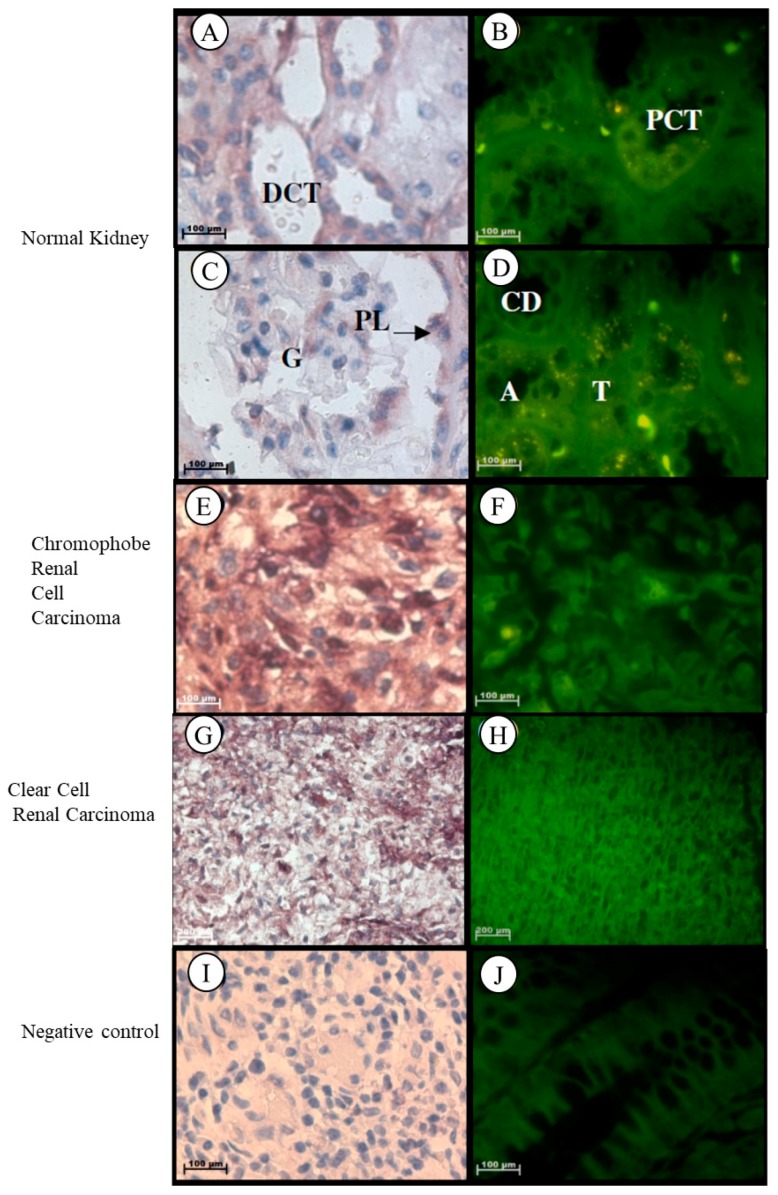
Subcellular Localization of *Tspo* mRNA in healthy and diseased Kidney Tissue: In healthy kidney tissue (**A**–**D**). *Tspo m*RNA is expressed in the cytoplasm of the flattened cells located in the parietal layer of the Bowman’s capsule, in the cytoplasm of endothelial cells that line the anastomosing network of capillaries of in the glomerulus (G), in the cytoplasm of the simple cuboidal epithelium that line the proximal convoluted tubule (PCT), in the cytoplasm of the simple squamous epithelium and erythrocytes within the vasa recta, in the cytoplasm of the simple squamous epithelium that lines the thin ascending and descending limbs (T), in the cytoplasm of the low cuboidal epithelium of the thick ascending limb (A), in the cytoplasm of the simple cuboidal epithelium of the distal convoluted tubule (DCT), in the cytoplasm of the simple cuboidal epithelium of the collecting tubule shape to the ascending limb and in the cytoplasm of the simple columnar epithelium that lines the collecting duct (CD). The transcription level of *Tspo* mRNA decreases in renal cell carcinoma (**F**,**H**) versus healthy kidney tissue (**B**). In Grade III chromophobe renal cell carcinoma (**E**,**F**) the mRNA is localized in the nuclei and cytoplasm of tumour cells that are usually arranged as a solid pattern with concentrations of the largest cells around the blood vessels. In renal clear cell carcinoma (**G**,**H**) the mRNA localizes in the nuclei and cytoplasm of tumour cells and in the cytoplasm of the delicate branching vasculature that appears fibromuscular. (**I**,**J**) Negative control for the subcellular localization of *Tspo* mRNA. Sense probes were used to probe tissue preparations and this was analysed colorimetrically and fluorescently. Tissues are magnified at 1000×.

Localization of the *Tspo* mRNA in normal kidney tissues was found in the cytoplasm of the following structures: flattened cells located in the parietal layer of the Bowman’s capsule, endothelial cells that line the anastomosing network of capillaries in the glomerulus, simple cuboidal epithelium with a prominent brush border of the tall microvilli that line the proximal convoluted tubule (PCT), simple squamous epithelium and erythrocytes within the vasa recta, characterized by an irregular shape, simple squamous epithelium that line the thin ascending and descending limbs characterized by a regular round shape, low cuboidal epithelium of the thick ascending limb that appears round in cross section, simple cuboidal epithelium of the distal convoluted tubule (DCT) characterized by the absence of a brush border and a larger more clearly defined lumen than the PCT, simple cuboidal epithelium of the collecting tubule that appear wider and less regular in shape than the ascending limb, and simple columnar epithelium that line the collecting duct (Figure 6A–D). *Tspo* mRNA was localized in the nuclei and cytoplasm of tumour cells that are usually arranged as a solid pattern with concentrations of the largest cells around the blood vessels of Grade III chromophobe renal cell carcinoma (Figure 6E,F). In clear cell renal carcinoma, *Tspo* mRNA was observed in the nuclei and cytoplasm of tumour cells and in the cytoplasm of the delicate branching vasculature that appear fibromuscular (Figure 6G,H).

#### 2.1.7. Breast

**Figure 7:**
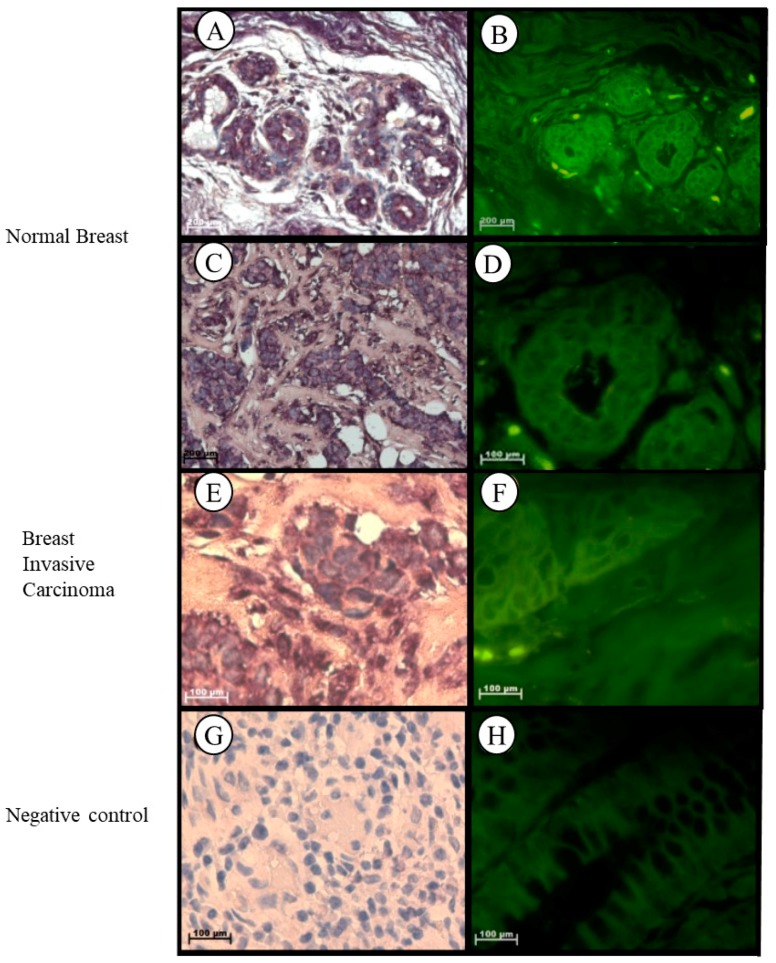
Subcellular Localization of *Tspo* mRNA in healthy and diseased Breast Tissue: *Tspo* mRNA is present in both healthy breast tissue (**A**,**B**) and Grade III invasive carcinoma (NST) (**C**,**D**). In normal breast tissue (**A**–**D**) *Tspo* is localized to the cytoplasm of the luminal layer of cuboidal epithelial cells and the outer layer of discontinuous epithelial cells of the terminal ducts (T) and alveoli (A), in cytoplasm of collagen fibres of the fibroconnective tissue, in nuclei and cytoplasm of lymphocytes and in the nuclei of endothelial cells of the vascular tissue located in the intralobular stroma. In Grade III invasive carcinoma (NST) (**E**,**F**) *Tspo* mRNA localizes to the cytoplasm of tumour cells arranged as nests and collagen fibres of the fibrotic stroma and in the nuclei and cytoplasm of lymphocytes located between the tumour and stroma, indicating the presence of a mononuclear infiltrate. (**G**,**H**) Negative control for the subcellular localization of *Tspo* mRNA. Sense probes were used to probe tissue preparations and this was analysed colorimetrically and fluorescently. Tissues are magnified at 1000×.

In normal breast tissue, *Tspo* mRNA was observed in the cytoplasm of the luminal layer of cuboidal epithelial cells, in the outer layer of the discontinuous epithelial cells of the terminal ducts and alveoli, in the collagen fibres of the fibroconnective tissue, in the nuclei and cytoplasm of lymphocytes, and in the nuclei of endothelial cells of the vascular tissue located in the intralobular stroma (Figure 7A–D). Localization of the *Tspo* mRNA in Grade III invasive carcinoma (NST) was observed in the cytoplasm of tumour cells arranged as nests and collagen fibres of the fibrotic stroma and in the nuclei and cytoplasm of lymphocytes located between the tumour and stroma, indicating the presence of a mononuclear infiltrate (Figure 7E,F).

#### 2.1.8. Brain

**Figure 8:**
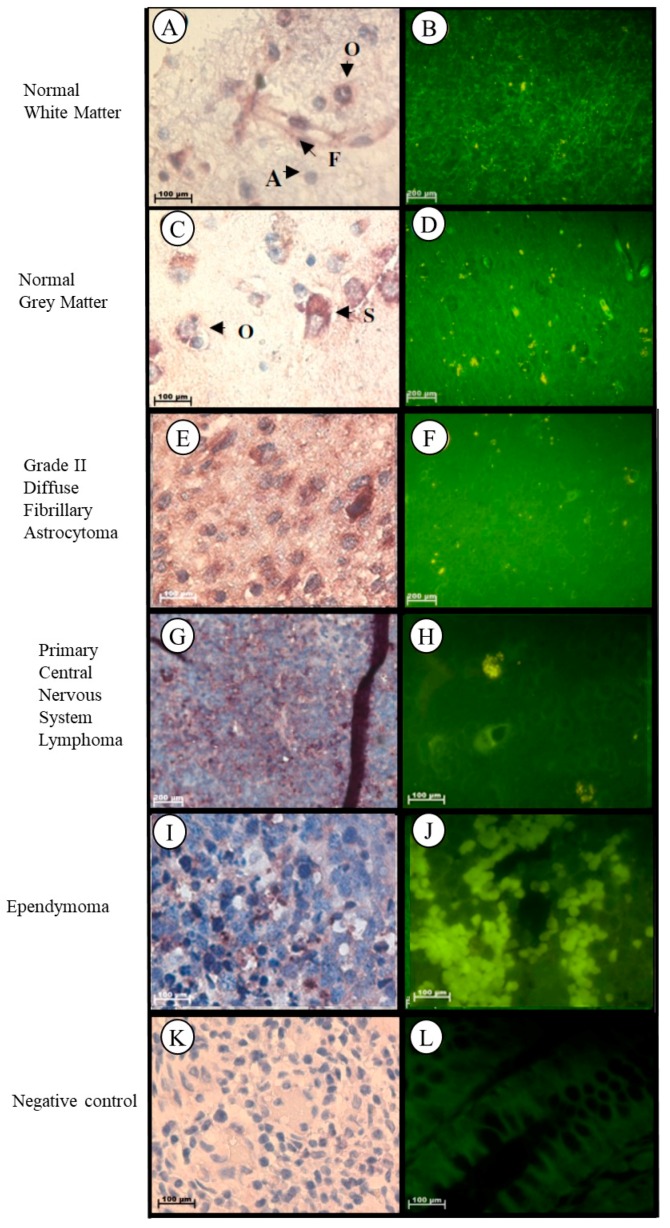
Subcellular Localisation of *Tspo* mRNA in healthy and diseased Brain Tissue. *Tspo* mRNA transcription occurs in both white (**A**,**B**) and grey (**C**,**D**) matter. Where it is found in astrocytes (A), fusiform cells (F), oligodendrocytes (O), and stellate cells (S). A comparison of the fluorescent intensity between normal (**B**,**D**) and cancer (**D**,**F**,**H**,**I**) tissue shows that *Tspo* transcription levels are highest in cancer tissues. *Tspo* mRNA is found in Grade II diffuse fibrillary astrocytoma (**E**,**F**), primary central nervous system lymphoma (PCNSL) (**G**,**H**) and ependymoma (**I**,**J**). Negative control for the subcellular localisation of Tspo mRNA (**K**,**L**). Sense probes were used to probe tissue preparations and this was analysed colorimetrically and fluorescently. Tissues are magnified at 1000×.

Localization of the *Tspo* mRNA in normal brain tissue was found in the cytoplasm of the following structures: neurons such as pyramidal cells, fusiform cells, in glia such as oligodendrocytes, and in the astrocytes and fibrillary network of white matter (Figure 8A,B). In grey matter *Tspo* mRNA is found in the glia such as oligodendrocytes, neurons such as stellate cells, astrocytes, and in the fibrillary network (Figure 8C,D). In Grade II diffuse fibrillary astrocytoma, *Tspo* mRNA was observed in the cytoplasm of the following structures: tumour cells that appear as glial cells having an oval shape appearance, neurons such as stellate cells, and oligodendrocytes, in the astrocytic processes that appear as fibrillary background, in the nuclei of microglia, and in the nuclei and cytoplasm of fusiform cells (Figure 8E,F). *Tspo* mRNA was localized in the cytoplasm of tumour cells that appear lymphocyte-like, in neurons such as oligodendrocytes and in the nuclei and cytoplasm of astrocytes, microglia, and fusioform cells of primary central nervous system lymphoma (PCNSL) (Figure 8G,H). Localization of the *Tspo* mRNA in ependymoma was observed in the nuclei and cytoplasm of fibrillary processes and tumour cells characterized by round to oval nuclei and an abundant granular cytoplasm, and in the nuclei of lymphocytes (Figure 8I,J).

### 2.2. The Level of Tspo mRNA Transcription Is Different in Healthy and Cancerous Tissue

**Figure 9:**
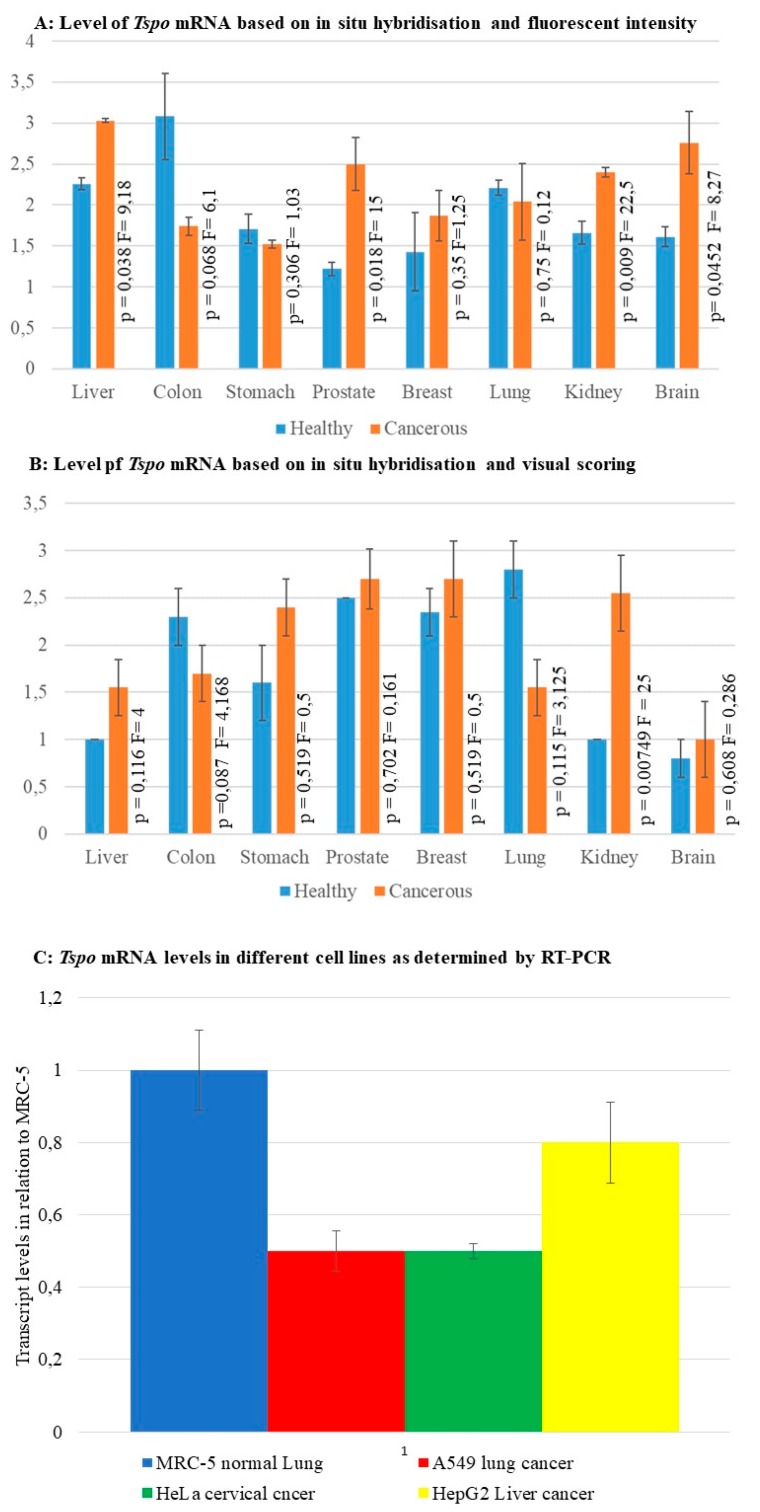
Relative transcription levels of *Tspo* based on different techniques (**A**) The levels of *Tspo* mRNA based on fluorescent intensity. (**B**) The levels of *Tspo* mRNA based on immunhistochemistry and visual scoring the levels of *Tspo* mRNA are significantly higher in liver, prostate, and brain cancer. At the same time the mRNA levels are lower in colon and lung cancer. (**C**) The levels of *Tspo* mRNA in different cell lines based on RT-PCR. The normal lung cell line MRC5 show the highest level of *Tspo* transcription, nearly twice that observed in the lung cancer cell line A549. The liver cancer cell line HepG2 had higher levels of *Tspo* transcription than lung cancer cells but lower than that of the normal lung cells.

Fluorescent intensity and visual scoring of fluorescence in situ hybridization staining as well as RT-PCR were used as a measure of *Tspo* transcription levels. The relative level of *Tspo* mRNA transcription was different when some cancer tissues were compared to their normal counterpart within a given organ. Both fluorescent and colorimetric methods showed an increase in *Tspo* transcription in renal cancers (Figure 6 and Figure 9A,B). Fluorescent staining (Figure 9A) showed a significant increase in the transcription of *Tspo* in liver (*p* = 0.038) (Figure 1B,D,F,H) prostate ((*p* = 0.018) Figure 4B,D,F,H) and brain cancer (*p* = 0.0452) (Figure 8B,D,F,H,J,L). Colorimetric staining showed an increase in *Tspo* transcription in the same tissues as fluorescent staining but these differences were not significant. Both fluorescent and colorimetric methods showed a decrease in *Tspo* transcription in both colon cancer (Figure 3 and Figure 9A,B) and lung cancer (Figure 5 and Figure 9A,B). Neither of these decreases were significant using either fluorescent or colorimetric analysis of in situ hybridisation (ISH). However, the decrease in *Tspo* levels observed in lung cancer was confirmed using RT-PCR analysis of *Tspo* mRNA levels in cancer (A549) and normal lung (MRC-5) cell lines (Figure 9C). The increase in *Tspo* transcription in liver cancer is supported by RT-PCR analysis of *Tspo* mRNA levels in the HepG2 liver cancer cell line, which were higher than A549 cells but lower than MRC5 lung cells. A summary of the changes in *Tspo* mRNA transcription that occur in cancer from different tissues is given in Table 1. 

## 3. Discussion

This study focused on determining the transcription pattern of *Tspo* mRNA in various types of human normal and cancer tissues by in situ hybridization. Although, TSPO expression has been primarily studied at the protein level, a previous study showed that *Tspo* mRNA is transcribed in all tissues, directly correlates with the reported protein expression levels, and suggested that the differential TSPO protein expression seen in the different tissues may be due at least in part to differences in transcriptional regulation [39]. Therefore, in this study, the level of *Tspo* mRNA transcription seen in the different tissues examined would need to allow for the relative correlation of TSPO protein expressed.

*Tspo* mRNA transcription was observed in all human normal and cancer tissues examined. As expected, *Tspo* mRNA localization was observed to mainly occur in the cytoplasm where translation to the TSPO protein occurs. However, nuclear localization of the *Tspo* mRNA occurred in some cases. This nuclear localization of *Tspo* mRNA seen in certain cells may be due to an increase in redox oxygen species (ROS) production that results in the nuclear accumulation of the Sp1 transcription factor and subsequent increase in transcription of *Tspo* mRNA [40]. A shift from homogenous cytoplasmic expression of TSPO to nuclear and perinuclear expression is observed in melanoma as it progresses to more advanced stages [37].

A cell-type specific transcription pattern of *Tspo* mRNA was observed. Consistent with previous studies [41], which demonstrated the presence of TSPO protein in the prostate [23,26,42,43,44], brain [38,44,45,46,47,48], stomach [23,44], colon [23,29,44,49], liver [23,44,50,51,52], lung [23,44,53], breast [23,54], and kidney [21,55,56]. *Tspo* mRNA transcription was observed to occur in most epithelial cells of the prostate, glia, and neurons of the brain, parietal cells, chief cells, and in the surface and neck mucous cells of the gastric glands of the stomach, hepatocytes, endothelial cells of the sinusoids, and Kupffer cells of the liver, most of the epithelial cells, goblet cells, and absorptive cells of the colon, most of the epithelial cells and macrophages of the lung, most of the epithelial cells of breast, most of the epithelial cells of the PCT, vasa recta, thin ascending and descending limbs, thick ascending limb, DCT, collecting tubule, collecting duct, and in the endothelial cells of the glomerulus of the kidney. The presence of *Tspo* mRNA in epithelial cells seen in the different organs further supports the role of the TSPO protein in differentiation.

Previous studies have shown that there is an increase in TSPO protein expression in differentiated cells compared to undifferentiated cells in different cell lines [57,58,59], melanoma cells [60], skin [61], and in many glandular epithelia such as the brush borders and microvilli found in the small intestine, colon, and stomach [23]. Moreover, the presence of the *Tspo* mRNA and TSPO protein seen in the glandular tissue of the stomach, colon, liver, and lung suggests that the TSPO protein may play a role in absorption and secretion through its effects on increased Ca^2+^ efflux [62]. High transcription of the *Tspo* mRNA and expression of the TSPO protein have been observed in mouse and human adipocyte differentiation [63,64]. Consistent with this, our study showed the presence of *Tspo* mRNA in the adipose cells located in the epiploon.

The relative level of *Tspo* mRNA transcription is different when cancer tissues were compared to its normal counterpart within many of the organ types. Consistent with previous studies a relative increase in *Tspo* mRNA transcription was observed in Grades II and III prostate adenocarcinoma of the peripheral duct and acini [26,42,65] and Grade II brain diffuse fibrillary astrocytoma [24]. In our study, only fluorescent detection of immunohistochemistry (IHC) staining indicated a significant increase in *Tspo* transcription in liver cancer, prostate cancer, and brain cancer. This would indicate a limited sensitivity in the use of *Tspo* mRNA as a biomarker for these cancers. However, radiolabelled TSPO ligands have shown high sensitivity for detecting the levels of TSPO protein in prostate [42] and brain cancer [45]. Another study indicated that TSPO is a poor biomarker for the diagnosis and prognosis of liver cancer, despite its upregulation in hepatocellular carcinomas [50]. Despite some previous studies indicating a significant decrease in *Tspo* mRNA transcription in renal clear cell carcinoma when compared to its normal counterpart [27], our study indicated a significant increase in *Tspo* transcription using both colorimetric and fluorescent analysis of ISH. Previous studies have indicated an increase in *Tspo* mRNA in Grade III breast invasive carcinoma (NST) [30,31,54,66], however, our study did not indicate any significant changes in the level of *Tspo* transcription between healthy and cancerous breast tissue. Previous studies also showed an increase in TSPO protein in colonic adenocarcinoma compared to its normal counterpart [27,29]; however, in our study we found no significant difference in *Tspo* mRNA transcription in colon cancer, when compared to its normal counterpart. These differences in expression may be attributed to differences in the nature of the studies. A decrease in the mRNA levels in lung and colon cancers may imply different energy metabolism or altered apoptosis regulation in these cancers. 

In conclusion, the widespread presence of the *Tspo* mRNA and TSPO protein in epithelial cells seen in the different organs suggests that the TSPO protein also plays an important role in non-steroidogenic tissues. Recently, there has been much debate concerning the roles played by TSPO in steroidogenesis [14,15]. TSPO plays a vital role in the processes of apoptosis, cell proliferation, and stress response [67]. It is known that epithelial cells can respond to stress and injury caused by ischemia, chemicals, and infection by rapidly proliferating and restoring the integrity of the epithelium [68]. The role played by TSPO in response to inflammation has led to the use of TSPO ligands as a biomarker for brain inflammation [13]. Our results further suggested that modulation of the regulation of *Tspo* mRNA transcription affects the expression of the TSPO protein in the different organs of the body as well as its expression in different cancer tissues [69]. Therefore, establishing the mechanisms that induce proliferation and restoration of the epithelial tissue and regulate *Tspo* mRNA transcription may help in understanding the role that the TSPO protein plays in various cancers and may allow for it to be exploited as a prognostic marker in cancers [31,70]. However, our results also suggest that the use of RNA probes to detect *Tspo* mRNA levels will be of limited use in most cancers as a biomarker for prognostic or diagnostic purposes. Brain, kidney, prostate, and liver cancer may be exceptions to this as the large increase in transcription was detected using in situ hybridization. While the use of RT-PCR to establish the levels of *Tspo* mRNA would be far more useful, the best biomarker would be the detection of the TSPO protein using radiolabelled ligands. 

## 4. Materials and Methods 

### 4.1. Human Tissue Arrays

Ethics approval to perform this study was obtained from the University of the Witwatersrand Research Ethics Committee (Medical), Johannesburg, South Africa (Protocol Number: M050223, 28 May 2004). Formalin-fixed tissue arrays containing multiple organs (liver, colon, prostate, breast, brain, lung, kidney, stomach, and epiploon) were purchased from Cybrdi™ Human-Derived Biological Products by Cybrdi, Inc. (Gaithersburg, MD, USA, catalogue number: CC00-11-002). Each of the tissue arrays contained 48 dots in the array panel, which represented a normal or cancer tissue spot from a specimen that was selected and pathologically confirmed histologically through H and E staining (Table 1). The array dot diameter was 1.5 mm and section thickness was 5 µm. The total number of cases on this tissue array was obtained from 46 individual patients (both normal and diseased combined). The experiment was done in triplicate for both colorimetric and fluorescent in situ hybridization.

### 4.2. Cell Culture

The HEK-293 cell line (American Type Culture Collection (ATCC), Manassas, VA, USA, catalogue number: ATCC^®^ CRL-1573™), which is a human embryogenic kidney cell line, was maintained in complete Dulbecco’s Modified Eagle’s Growth Medium (DMEM) (Gibco^®^ by Invitrogen by Life Technologies by Thermo Fisher Scientific, Carlsbad, CA, USA) supplemented with 10% (*v*/*v*) foetal bovine serum (FBS) (Gibco^®^), 1mM sodium pyruvate (Gibco^®^), 1 mM MEM non-essential amino acids (Gibco^®^) and 2% (*v*/*v*) 100X penicillin streptomycin glutamine (Gibco^®^). All other cell lines used were maintained in complete growth Dulbecco’s Modified Eagle’s Medium (DMEM) (Gibco^®^ Carlsbad, CA, USA) and 2% (*v*/*v*) 100X penicillin streptomycin glutamine (Gibco^®^) supplemented with 10% (*v*/*v*) foetal bovine serum (FBS) (Gibco^®^). These included the normal human embryonic fibroblast like lung cell line, MRC5, the human epithelial cell lung carcinoma cell line, A549, the human liver hepatocellular carcinoma cell line, HepG2, and the human epithelial cell cervical carcinoma cell line, HeLa. All cells were incubated at 37 °C in a humid incubator containing 5 % (*v*/*v*) CO_2_. These cells were passaged every 2 to 3 days and harvested when confluent. All these cell lines were purchased from Highveld Biological (Pty) Ltd. (Johannesburg, South Africa). These cells were used to extract RNA in order to synthesize the *Tspo* probe as well as to establish *Tspo* transcription levels in different cancer cells.

### 4.3. RNA Probe Synthesis

Total RNA was harvested from HEK-293 cells using the Trizol™ LS Reagent (Ambion by Life Technologies, Thermo Fisher Scientific, Carlsbad, CA, USA) following the manufacturer’s protocol. Thereafter, an aliquot of 2 µL was reverse transcribed to synthesize cDNA using a First Strand cDNA Synthesis Kit (Roche Diagnostics GmbH, Manneheim, Germany) following the manufacturer’s protocol. Subsequently, the cDNA was used as a template to amplify part of the *Tspo* mRNA using primers specific for the *Tspo* gene (primers were designed using the following sequence as template: Accession Number: BT006949; TSPO F-5′-TTCACAGAGAAGGCTGTGGTTC-3′ and TSPO R-5′-GCCATACGCAGTAGTTGAGTGT-3′) resulting in a 247 bp DNA fragment. PCR was performed in GeneAmp^®^ PCR System 9700 thermocycler (Applied Biosystems, Foster City, CA, USA) using the following reaction components: 1X PCR Master Mix (Promega Corporation, Madison, WI, USA), 1 µM forward primer, 1 µM reverse primer, 1.5 mM MgCl_2_, and 1.0 µL cDNA, with an initial denaturation at 95 °C for 2 min followed by 30 cycles of denaturation at 95 °C for 30 s, annealing at 58 °C for 30 s, and elongation at 72 °C for 1 min with a final elongation at 72 °C for 1 min. The PCR product was ligated into the pGEM-T Easy vector (Promega Corporation) using the LigaFast™ Rapid Ligation System (Promega Corporation) following the manufacturer’s protocol. The resultant plasmid constructs were transformed and propagated in chemically competent MC1061 *Escherichia coli* cells (Lucigen Corporation, Middleton, WI, USA). Sequencing with the T7 F and SP6 R primers confirmed the presence and orientation of the TSPO DNA. The sequence confirmed plasmid DNA was then linearized with *Pst*I or *Apa*I in preparation for the generation of the anti-sense and sense RNA probes, respectively. Linearized fragments were transcribed in vitro with T7- or SP6-RNA polymerase for the anti-sense and sense probe, respectively, and labelled with digoxigenin (DIG) using the DIG RNA Labelling Kit (SP6/T7) (Roche Diagnostics GmbH, Manneheim, Germany), following the manufacturer’s protocol. 

### 4.4. In Situ Hybridization

The tissue arrays were pre-treated by dewaxing in fresh xylene, followed by rehydration by washing them sequentially in decreasing (*v*/*v*) concentrations of ethanol (100%, 90%, 70%, and 50%) and finally they were fixed with 4% (*w*/*v*) paraformaldehyde for 20 min. Protein denaturation in sections was performed with 0.1 M HCl for 10 min. This was followed with treatment with 0.5% (*v*/*v*) acetic anhydride for 10 min to limit non-specific labelling and incubation with 10 µg/µL Proteinase K (Promega Corporation) at 37 °C for 20 min to permeabilize the cell membrane, the Proteinase K activity was then terminated and the tissue array sections were dehydrated in increasing (*v*/*v*) concentrations of ethanol (50%, 70%, 90%, and 100%). Finally the array was dried in chloroform. DIG-labelled anti-sense RNA probes and DIG-labelled sense RNA probes were prepared by dissolving the appropriate amount of probe in Hybridization Buffer (Roche Diagnostics GmbH) containing 0.01 µg/µL Herring Sperm DNA (Promega Corporation). The probes were incubated at 100 °C for 5 min, followed by incubation on ice for 2 min. The tissue array sections were incubated overnight at 55 °C with the freshly prepared probes and covered with a solution containing 50% (*v*/*v*) formamide and 5% (*v*/*v*) sodium chloride sodium citrate solution (SSC). The following day, the tissue array sections was washed in 2X SSC for 30 min at 37 °C and sequentially for 20 min at 55 °C with 1X-, 0.5X- and 0.1X SSC. Thereafter, the tissue array sections were washed three times, 1 min per wash with Tris-buffered saline (TBS) followed by blocking with 1X Blocking Solution (Roche Diagnostics GmbH) for 15 min. Tissue sections hybridized with the sense probe served as the negative control.

In order to perform colorimetric detection, the tissue sections were incubated for 1 h with 1:50 anti-DIG IgG diluted in 1X Blocking Buffer followed by washing three times, 1 min per wash with TBS. Subsequently, the tissue array sections were incubated and left to develop overnight with 1:50 nitroblue tetrazolium/5-bromo-4-chloro-3-indolyl phosphate (NBT/BCIP) diluted in 1X Detection Buffer (both from Roche Diagnostics GmbH). The following day, the reaction was terminated by incubating the tissue array sections with 1X Tris-EDTA for 5 min followed by rinsing with water for 5 min. Thereafter, the tissue array sections were counterstained for 1 min with Mayer’s haematoxylin (Sigma-Aldrich Inc., St. Louis, MO, USA) followed by rinsing with water for 10 min. The tissue sections were mounted with aqueous glycerol jelly and allowed to dry at 37 °C for 1 h after which they were viewed and analysed using a light microscope.

In order to perform fluorescent detection, the tissue array sections were washed with TBS–Tween for 5 min followed by incubation at 37 °C for 30 min with Tri-Sodium Blocking Solution (TNB) (Roche Diagnostics GmbH). Subsequently, the tissue array sections were incubated at 37 °C for 30 min with anti-DIG fluorescein isothiocyanate (FITC) followed by washing three times, 5 min per wash with TBS-Tween. The tissue sections were mounted with Molecular Probes SlowFade^®^ Light Antifade Kit (Molecular Probes, Eugene, OR, USA), following the manufacturer’s protocol after which they were viewed and analysed using a fluorescent microscope with a 490 nm filter.

### 4.5. Image and Statistical Analysis

Images were captured using the AxioCam (MRm/MRc) camera and AxioVision software package (Carl Zeiss Microimaging GmBH by Carl Zeiss AG, Oberkochen, Germany). The fluorescent intensity produced by the fluorescently labelled in situ hybridisation probes was measured and used as an indicator of the level of *Tspo* mRNA. The levels of fluorescence were normalised using the negative controls. Significant differences were determined using one-way analysis of variance (ANOVA) followed by Tukey’s multiple comparison test. A probability level of *p* < 0.05 was considered significant. The intensity of the colorimetric staining was assessed visually by assigning a score to each sample based on the level of staining observed in the malignant cells. A score of 0 represented no staining, a score of 1 represented <10% of cells were stained, a score of 2 represented 10–50% of cells were stained and finally, a score of 3 represented >50% of cells were stained. Significant differences were determined using one-way analysis of variance (ANOVA) followed by Tukey’s multiple comparison test. A probability level of *p* < 0.05 was considered significant.

### 4.6. Real-Time Polymerase Chain Reaction

Reverse transcription was performed using an ImProm-II^TM^ Reverse Transcription System (Promega Corporation) with a MgCl_2_ concentration of 5 mM using Oligo dt (15) primers. Real-Time PCR was carried out in a total volume of 25 μL using the IQ^Tm SYBR^ Green mix (Bio Rad Laboratories, Hercules, CA, USA). The forward primer 5′-TTCACAGAGAAGGCTGTGGTTC-3′ and the reverse primer 5′-GCCATACGCAGTAGTTGAGTGT-3′ were used at a concentration of 0.4 pmol/µL. An initial 120 s denaturation step was performed followed by forty cycles of amplification were carried out consisting of a 30 s 95 °C denaturation step followed by a 30 s primer annealing step carried out at 58 °C and a final 60 s extension step carried out at 72 °C. After 40 cycles of amplification a final 600 s extension step was performed. A no template negative control was included. Results were analysed by quantitation and melting curves using the Opticon 3.1 software (Bio-Rad Laboratories, Hercules, CA, USA).

## 5. Conclusions

Despite most cancers showing an increase in the transcription of ***Tspo*** mRNA and there being observable changes in the localisation of the mRNA, the levels of the mRNA itself would not necessarily make a good diagnostic or prognostic marker. In most cancers the change is small and not significant, while in those that the change is significantly different the use of ISH to identify changes in *Tspo* transcription woul still be more invasice and less accurate than the use of labelled TSPO ligands. 

## Figures and Tables

**Table 1:** Summary of localization and transcription data for *Tspo *in cancer from various tissues.

Prostate		
Tissue Type	Increase Decrease	Localisation
Normal		Cytoplasm of the inner columnar epithelium lining the glands Collagen and elastic fibres of the fibromuscular stroma Cytoplasm and nuclei of the outer endothelial cells that line the glands Nuclei of fibroblasts of the fibromuscular stroma
Grade II adenocarcinoma	–Insignificant increase	Found in same locations as in normal tissue, but also found in the collagen fibres of the fibromuscular stroma Nuclei and cytoplasm of lymphocytes and endothelial cells lining blood vessels of the fibromuscular stroma
Grade III adenocarcinoma		Found in same locations as in normal tissue, but also found in the cytoplasm of tumour cells that grow in nests or sheets of the peripheral duct and acini
**Stomach**		
Epiploon		Cytoplasm of adipose cells, Collagen fibres Fibroblasts located between the collagen fibres in the stroma
Stomach		Cytoplasm of parietal cells, chief cells Surface and neck mucous cells of the gastric glands Collagen fibres, plasma cells and macrophages located in the lamina propria Nuclei and cytoplasm of lymphocytes located in the lamina propria
Grade III stomach squamous cell carcinoma	–Insignificant increase	Tumour cells and collagen fibres in the surrounding stroma
**Colon**		
Normal		Cytoplasm of the goblet and absorptive cells of the Crypts of Lieberkhün, Plasma cells located in the lamina propria Nuclei and cytoplasm of lymphocytes located in the lamina propria of normal colon tissue
Grade I adenocarcinoma	–Significant decrease	Cytoplasm of tumour cells that are mucin-secreting and arranged in adenomatous tubular glands and fibromuscular stroma
Grade II adenocarcinoma		Cytoplasm of tumour cells arranged as adenomatous glands or solid sheets
Grade III adenocarcinoma		Cytoplasm of tumour cells that are predominantly arranged as a solid pattern
**Liver**		
Normal		Cytoplasm of hepatocytes Nuclei and cytoplasm of endothelial cells of the sinusoids and Kupffer cells
Grade II Hepato-cellular carcinoma	–Significant increase	Cytoplasm of tumour cells having either a trabecular or pseudoglandular pattern
Grade III Hepato-cellular carcinoma		Nuclei and cytoplasm of tumour cells that resemble anaplastic giant to spindle-shaped cells
**Lung**		
Normal		Cytoplasm of macrophages, plasma cells and fibroblasts in the surrounding stroma Cytoplasm of in the cuboidal cells that line the respiratory bronchiole Cytoplasm of in the smooth muscle fibres surrounding the pulmonary artery Nuclei and cytoplasm of the endothelial cells of the pulmonary artery and lymphocytes in the surrounding stroma of healthy lung tissue
Grade III lung adenocarcinoma	–Significant decrease	Cytoplasm and nuclei of tumour cells and in the fibres located in the surrounding solid-like stroma
Grade III lung squamous cell carcinoma		Cytoplasm and nuclei of tumour cells of that is characterized by the merging of tumour cells to form a large cell pattern
small cell carcinoma		Cytoplasm of tumour cells that resemble lymphocytes Nuclei of lymphocytes and endothelial cells lining capillaries in the stroma
**Kidney**		
Normal		Cytoplasm of the flattened cells located in the parietal layer of the Bowman’s capsule Cytoplasm of the endothelial cells that line the anastomosing network of capillaries in the glomerulus, Cytoplasm of the simple cuboidal epithelium that line the proximal convoluted tubule (PCT), Cytoplasm of the simple squamous epithelium and erythrocytes within the vasa recta Cytoplasm of the simple squamous epithelium that line the thin ascending and descending Cytoplasm of the low cuboidal epithelium of the thick ascending limb Cytoplasm of the simple cuboidal epithelium of the Distal Convoluted Tube Cytoplasm of the simple cuboidal epithelium of the collecting tubule Cytoplasm of the and simple columnar epithelium that line the collecting duct
Grade III chromophobe renal cell carcinoma	–Significant increase	Nuclei and cytoplasm of tumour cells that are usually arranged as a solid pattern with concentrations of the largest cells around the blood vessels
Clear cell renal carcinoma		Nuclei and cytoplasm of tumour cells Cytoplasm of the delicate branching vasculature
**Breast**		
Normal		Cytoplasm of the luminal layer of cuboidal epithelial cells Outer layer of the discontinuous epithelial cells of the terminal ducts and alveoli Collagen fibres of the fibroconnective tissue Nuclei and cytoplasm of lymphocytes Nuclei of endothelial cells of the vascular tissue located in the intralobular stroma
Grade III invasive carcinoma (NST)		Cytoplasm of tumour cells arranged as nests Collagen fibres of the fibrotic stroma Nuclei and cytoplasm of lymphocytes located between the tumour and stroma
**Brain**		
White matter		Cytoplasm of neurons such as pyramidal cells Cytoplasm of fusiform cells Cytoplasm of glia such as oligodendrocytes Cytoplasm of the astrocytes and fibrillary network
Grey matter		Glia such as oligodendrocytes Neurons such as stellate cells Astrocytes Fibrillary network
Grade II astrocytoma	–Significant increase	Cytoplasm of tumour cells that appear as glial Cytoplasm of neurons’ such as stellate cells and oligodendrocytes, Cytoplasm of astrocytic processes Nuclei of microglia Nuclei and cytoplasm of fusiform cells
CNS lymphoma		Cytoplasm of tumour cells that appear lymphocyte-like Neurons such as oligodendrocytes Nuclei and cytoplasm of astrocytes, microglia and fusioform cells of primary central nervous system lymphoma (PCNSL)
Ependymoma		Nuclei and cytoplasm of fibrillary processes and tumour cells Nuclei of lymphocytes

## Abbreviations

TSPO	translocator protein
CNS	central nervous system
VDAC	voltage-dependent anion channel
ANC	adenine nucleotide carrier
